# Phacoemulsification with Goniosynechialysis versus Phacoemulsification Alone in Angle-Closure Glaucoma: A Meta-Analysis of Randomized Controlled Trials

**DOI:** 10.1155/2021/8831479

**Published:** 2021-02-15

**Authors:** Ji-Guo Yu, Fang Zhao, Yi Xiang

**Affiliations:** Department of Ophthalmology, The Central Hospital of Wuhan, Tongji Medical College, Huazhong University of Science and Technology, Wuhan 430014, Hubei Province, China

## Abstract

**Purpose:**

This meta-analysis aimed to compare the efficacy and safety of phacoemulsification with goniosynechialysis (Phaco-GSL) with those of phacoemulsification alone (Phaco-alone) in patients with angle-closure glaucoma and cataract.

**Methods:**

Randomized controlled trials (RCTs) were selected through a search of electronic databases. Trial eligibility and risk of bias were assessed using Cochrane review methods. Primary measures included the intraocular pressure (IOP), number of antiglaucoma medications, peripheral anterior adhesion (PAS) extent, and their pre- and postoperative changes. For continuous parameters, we calculated weighted mean differences and 95% confidence intervals. Statistical analysis was performed using RevMan 5.3 software.

**Results:**

Eight RCTs were included, where 224 and 236 eyes were in the Phaco-GSL and Phaco-alone groups, respectively. Both postoperative IOP and number of medications were not significantly different between the study groups at the six- and twelve-month follow-up. However, the 12-month postoperative PAS extent was significantly smaller in the Phaco-GSL group than in the Phaco-alone group. Similarly, changes from the preoperative to 12-month postoperative PAS extent were significantly greater in the Phaco-GSL group than in the Phaco-alone group, but IOP and the number of medication changes were not different.

**Conclusions:**

Our results provide evidence that Phaco-GSL provides advantages over Phaco-alone treatment regarding PAS reduction. In terms of IOP and medication reduction, both groups were comparable. Thus, Phaco-GSL can be considered for the treatment of patients with angle-closure glaucoma and cataract due to its ease, safety, and potential benefit for the anterior chamber angle.

## 1. Introduction

Glaucoma is a group of diseases characterized by deformation and atrophy of the optic nerve head due to a rise in intraocular pressure (IOP), leading to visual field defects and visual impairment. It is the second most frequent blinding eye disease in the world and poses a serious threat to visual health in humans [1]. Glaucoma can be divided into many types. For angle-closure glaucoma, common causes are a shallow anterior chamber and extended range of permanent peripheral anterior adhesion (PAS), leading to a blocked outflow of aqueous humour and consecutive increase in IOP [2]. The thickened and anterior-positioned lens plays a crucial role in the development of angle-closure glaucoma. With the development of ageing and cataract, the lens progressively increases in size and thickness and gradually approaches the pupil edge, resulting in a pupillary block. This causes a continuous increase in posterior chamber pressure and further compression of the peripheral iris, leading to anterior chamber angle stenosis or even closure [3]. Therefore, cataract removal has been used to deepen the anterior chamber and open the iridocorneal angle, thereby reducing the IOP [4–6].

However, ophthalmologists have found that phacoemulsification alone (phaco-alone) may sometimes not be able to open the chamber angle sufficiently to control the IOP, so they suggested to combine this approach with goniosynechialysis (GSL) or even trabeculectomy [7–9]. Goniosynechialysis is a method to deepen the chamber angle in cataract surgery using a mechanical or viscoelastic agent [10, 11]. The operation is not difficult, and complications are less frequent than those observed in trabeculectomy. Some researchers found that the IOP was well-controlled after phacoemulsification with goniosynechialysis (Phaco-GSL), which was in some studies demonstrably better than Phaco-alone [12, 13]. However, some researchers suggest that Phaco-GSL has no obvious advantages over Phaco-alone [14, 15]. Whether to combine phacoemulsification with GSL for the treatment of patients with narrowed chamber angles and cataracts is a controversial and debated issue. Given these inconsistent viewpoints, we performed a meta-analysis of randomized controlled trials (RCTs) to clarify whether Phaco-GSL has advantages over Phaco-alone to treat patients with angle-closure glaucoma and cataract.

## 2. Methods

### 2.1. Search Strategy

This meta-analysis was conducted in accordance with the Cochrane Handbook for Systematic Reviews of Interventions and Preferred Items for Systematic Reviews and Meta-Analysis (PRISMA) Statement. A literature search of the PubMed, EMBASE, and Cochrane Library databases up to March 25, 2020, was performed to identify relevant studies. The following terms were used for this search: “primary angle-closure glaucoma,” “chronic angle-closure glaucoma,” “angle-closure glaucoma,” “primary angle closure,” “phacoemulsification,” “cataract extraction,” “cataract surgery,” “goniosynechialysis,” and “viscogonioplasty.” Results from the electronic databases were imported into a reference management program (EndNote X4; Thomson Reuters, New York, NY, USA), where duplicate articles were manually deleted. Afterwards, the titles and abstracts of all studies were independently perused by two authors (J.G.Y. and F.Z.). Subsequently, the full texts of the remaining potentially relevant reports were read to determine whether they met the inclusion criteria described in the following. In addition, the reference lists of these studies were also screened to identify any relevant studies that were not retrieved from the computerized databases.

### 2.2. Inclusion and Exclusion Criteria

Studies were considered for inclusion if they met the following criteria: (1) the study was an RCT; (2) the study compared Phaco-GSL with Phaco-alone; (3) the study examined angle-closure diseases including primary angle-closure glaucoma (PACG), primary angle-closure, and chronic angle-closure glaucoma (CACG); (4) the study used the surgical technique viscogonioplasty (VGP); and (5) the last follow-up time point of an enrolled study was three months or more. Exclusion criteria were as follows: (1) any study without a Phaco-alone group; (2) any study that included other surgeries such as trabeculectomy; (3) the relevant data of a study could not be used for the meta-analysis; and (4) retrospective studies, case reports, and review articles. Two reviewers (J.G.Y. and Y.X) separately evaluated the studies based on these inclusion and exclusion criteria, and discrepancies were resolved through discussion.

### 2.3. Data Extraction

Two reviewers (J.G.Y. and F.Z.) independently extracted data from each of the included studies. The following information was retrieved from each study: first author, year of publication, study location, study design, type of glaucoma, number of eyes, mean age, sex, technique, and follow-up period. The main outcome parameters of the current study included the IOP, number of antiglaucoma medications, PAS extent before surgery and at the follow-up time point, and the changes between their pre- and postoperative values. Since two studies assessed the PAS extent in clock hours [13, 16], we converted their values to circular degrees by multiplying the number of hours with 30. Any discrepancies between the reviewers' results were resolved via discussion with another author (Y.X.).

### 2.4. Quality Assessment

The Cochrane Risk of Bias Assessment Tool was applied to determine the risk of bias in evaluating the quality of the included RCTs. Seven domains concerning the quality of the RCTs were observed: (1) random sequence generation, (2) allocation concealment, (3) blinding of participants and personnel, (4) blinding of outcome assessment, (5) incomplete outcome data, (6) selective reporting, and (7) other bias. Each domain was graded into “low risk of bias,” “high risk of bias,” or “unclear risk of bias.” Two reviewers (F.Z. and Y.X.) independently evaluated the studies using this tool, and disagreements were resolved via discussion.

### 2.5. Statistical Analysis

Statistical analyses were performed using the RevMan software (version 5.3; Cochrane Collaboration, Oxford, UK). The main outcomes of this meta-analysis are continuous scale variables that are expressed as the mean ± standard deviation (SD). Summary estimates, including 95% confidence intervals (CIs), were calculated. For each continuous outcome parameter, the mean and SD were used to calculate the weighted mean difference (WMD). The heterogeneity of studies was assessed using the chi-squared test, with *P* < 0.05 and *I*^2^> 50% indicating significant heterogeneity [17]. Heterogeneity was considered low when *I*^2^ ≤ 50%, and the fixed-effects model was applied. For *I*^2^ > 50%, the random-effects model was used [18]. A *P* value < 0.05 was considered to be statistically significant.

## 3. Results

### 3.1. Study Selection

**Figure 1:**
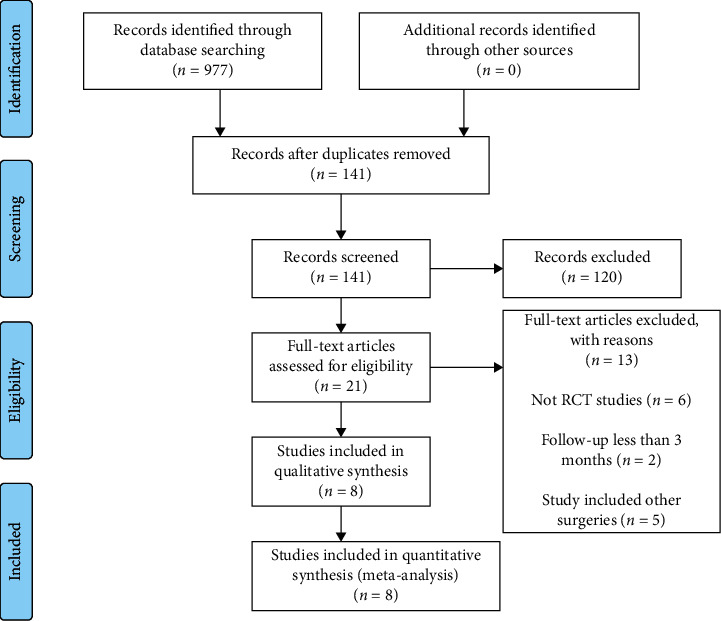
Flowchart of the literature search and selection.

A total of 977 records were identified through database searches. After duplicate removal, 141 records remained, of which 120 were excluded after reading the title and abstract. This resulted in a total of 21 reports warranting evaluation for eligibility by reading the full-text articles. Of these 21 reports, six studies were not RCTs, two studies had follow-up periods of less than three months, and five studies included other surgeries. These reports failed to meet the inclusion criteria of this study and were excluded from the meta-analysis. Thus, eight RCTs met our study criteria and were included in the final meta-analysis [12, 13, 15, 16, 19–22]. The study selection process is summarized in Figure 1.

### 3.2. Characteristics of the Enrolled Studies

**Figure 2:**
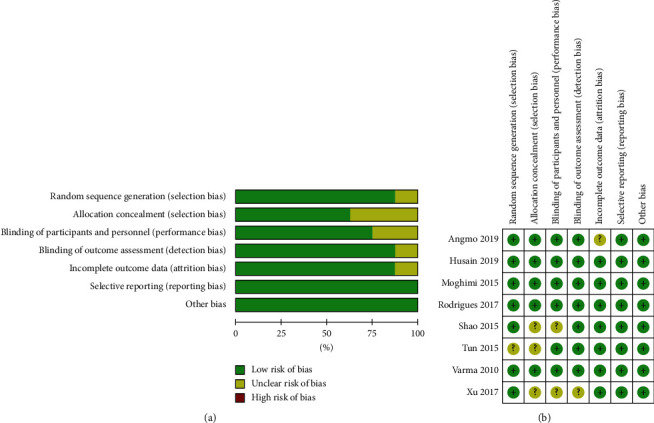
Assessment of the risk of bias in included studies. (a) Risk of bias graph. Judgments about each “risk of bias” item are presented as percentages across all included studies. (b) Risk of bias summary, describing the detailed risk of bias values for each article. +: low risk of bias; −: high risk of bias; ?: unclear risk of bias.

The included studies were published between 2010 and 2019 and comprised a total of 460 eyes, of which 224 were in the Phaco-GSL group and 236 in the Phaco-alone group. Two studies were performed in the United Kingdom [12, 21], two were performed in China [20, 22], one was a multicentre, international randomized clinical trial conducted at the four study sites of Singapore, Vietnam, Thailand, and Hong Kong [16], and one study each was performed in Iran [19], Singapore [13], and India [15]. The age of the patients ranged from 53 to 74 years. The number of eyes analysed in these studies ranged from 10 to 46. The baseline characteristics of each included study are shown in Table 1, and the risk of bias assessment is summarized in Figure 2. Overall, the included studies were at low risk of bias.

### 3.3. Outcome Measures of the Meta-Analysis

The main outcomes assessed in this meta-analysis included the IOP, number of antiglaucoma medications, and PAS extent at baseline and after surgery at six- and twelve-month follow-up and the changes in these parameters between preoperative and twelve-month postoperative values.

### 3.4. Preoperative and Postoperative IOP Values

**Figure 3:**
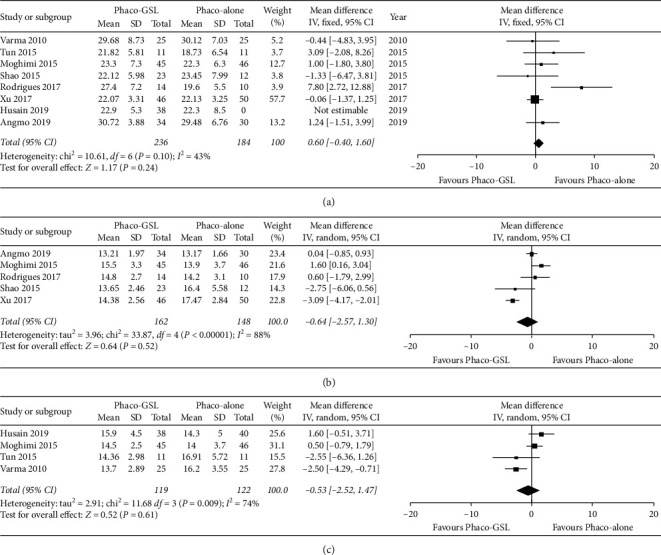
Forest plots comparing the IOP between the Phaco-GSL and Phaco-alone groups before and after surgery at different follow-up times. (a) Before surgery; (b) six months postoperatively; (c) twelve months postoperatively.

The preoperative IOP was compared between the Phaco-GSL and Phaco-alone groups across eight studies. The meta-analysis of these data demonstrated that the mean IOP values were not significantly different between the two groups (WMD = 0.6, 95% CI: −0.40 to 1.60, *P*=0.24; Figure 3(a)).

The postoperative IOP values at the six- and twelve-month follow-up were compared across five and four studies, respectively, between the Phaco-alone and Phaco-GSL groups. The meta-analysis of these data revealed that the IOPs were not significantly different between the two groups at six months postoperatively (WMD = -0.64, 95% CI: −2.57 to 1.30, *P*=0.52; Figure 3(b)) or at twelve months postoperatively (WMD = −0.53, 95% CI: −2.52 to 1.47, *P*=0.61; Figure 3(c)).

### 3.5. Preoperative and Postoperative Numbers of Medications

**Figure 4:**
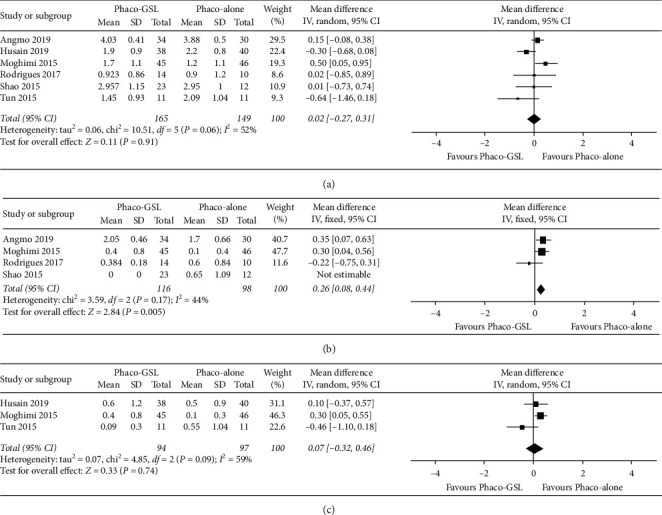
Forest plots comparing the number of medications between the Phaco-GSL and Phaco-alone groups before and after surgery at different follow-up times. (a) Before surgery; (b) six months postoperatively; (c) twelve months postoperatively.

The preoperative number of antiglaucoma medications was compared between the Phaco-GSL and Phaco-alone groups across six studies. No significant difference in the number of medications used to reduce the IOP was detected in the meta-analysis comparing the two groups (WMD = 0.02, 95% CI: −0.27 to 0.31, *P*=0.91; Figure 4(a)).

The postoperative number of medications at the six-month follow-up was reported by four of the enrolled studies. The meta-analysis of these data showed that the number of medications in the Phaco-alone group was significantly lower than that in the Phaco-GSL group (WMD = 0.26, 95% CI: 0.08 to 0.44, *P*=0.005; Figure 4(b)).

Additionally, the postoperative number of antiglaucoma medications was compared between the Phaco-GSL and Phaco-alone groups at the twelve-month follow-up across three studies, but meta-analysis of these data showed that the two groups did not significantly differ in the number of medications (WMD = 0.07, 95% CI: −0.32 to 0.46, *P*=0.74; Figure 4(c).

### 3.6. Preoperative and Postoperative PAS Extent

**Figure 5:**
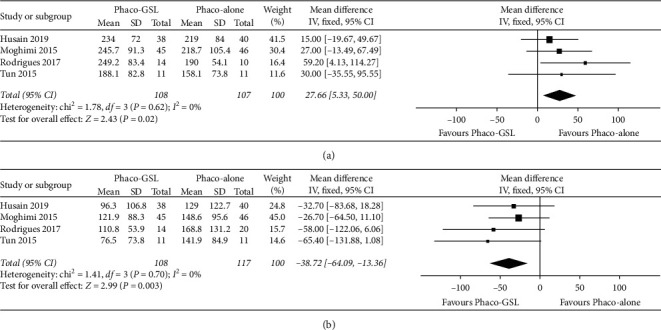
Forest plots comparing the PAS extent between the Phaco-GSL and Phaco-alone groups before and after surgery at different follow-up times. (a) Before surgery; (b) twelve months postoperatively.

The preoperative PAS extent was compared between the Phaco-GSL and Phaco-alone groups across four studies. The meta-analysis of these data showed that the extent of PAS in the Phaco-GSL group was significantly greater than that in the Phaco-alone group (WMD = 27.66, 95% CI: 5.33 to 50.00, *P*=0.02; Figure 5(a)).

Due to lack of data, we did not analyse the postoperative PAS extent at the six-month follow-up, but the postoperative PAS extent at the twelve-month follow-up was compared between the Phaco-GSL and Phaco-alone groups across three studies. The meta-analysis of these data demonstrated that the PAS extent was significantly smaller in the Phaco-GSL group than in the Phaco-alone group (WMD = −38.72, 95% CI: −64.09 to −13.36, *P*=0.003; Figure 5(b)).

### 3.7. Changes from Preoperative to 12-Month Postoperative Conditions

**Figure 6:**
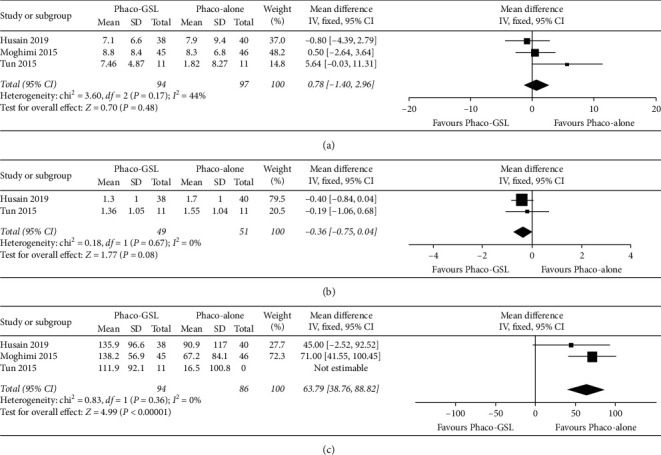
Forest plots of changes in IOP, number of medications, and PAS extent from preoperative to 12-month postoperative values comparing the Phaco-GSL and Phaco-alone groups. (a) IOP; (b) number of medications; (c) PAS extent.

The change from preoperative to 12-month postoperative IOP was compared between the Phaco-GSL and Phaco-alone groups across three studies. The meta-analysis of these data showed that the reduction in IOP was not significantly different between the two groups (WMD = 0.78, 95% CI: −1.40 to 2.96, *P*=0.48; Figure 6(a)).

The change in the number of medications from preoperative to 12-month postoperative values was compared between the Phaco-GSL and Phaco-alone groups across the two studies. Meta-analysis of these data confirmed that the reduction in the number of medications was not significantly different between the two groups (WMD = −0.36, 95% CI: −0.75 to 0.04, *P*=0.08; Figure 6(b)).

Furthermore, the change from preoperative to 12-month postoperative PAS extent was compared across three studies. The meta-analysis demonstrated that the change in PAS extent was significantly greater in the Phaco-GSL group than in the Phaco-alone group (WMD = 63.79, 95% CI: 38.76 to 88.82, *P* < 0.00001; Figure 6(c)).

## 4. Discussion

It is well known that pupillary block caused by the opacity of the lens plays an important role in the pathogenesis of angle-closure glaucoma. In the general population, angle-closure glaucoma is most common in people over the age of 50 years and is often associated with cataracts. Removal of the lens can eliminate the pupil block, substantially improve the congestion of the anterior segment, deepen the anterior chamber, widen the chamber angle or reopen it, and effectively prevent the development of angle-closure glaucoma [23]. Therefore, phacoemulsification and implantation of a foldable intraocular lens (IOL) is a viable operation for the treatment of angle-closure glaucoma. This surgical procedure can decrease the IOP and number of required antiglaucoma medications [24, 25]. However, many ophthalmologists have adopted Phaco-GSL to treat patients with angle-closure glaucoma, and they believe that this approach can better remove any present PAS to open the chamber angle and reduce the IOP.

Previous studies have shown that the postoperative IOP can be well-controlled by phacoemulsification and IOL implantation alone for patients with angle-closure glaucoma accompanied by PAS less than 180°, whereas, for patients with excessive chamber angle adhesion, GSL should be considered to separate these adhesions [15]. However, we believe that, for patients with PAS less than 180°, we should also routinely combine phacoemulsification with GSL to separate the PAS as much as possible, to protect the drainage function of the chamber angle, and to avoid the possibility of continued adhesion exceeding 180° that ultimately may increase the IOP. For patients with a larger PAS range, that is, more than 180°, Phaco-GSL should also be used as a first-line treatment. Prolonged PAS and chamber angle closure may lead to irreversible damage of the trabecular meshwork, including trabecular collapse and scarring, and result in failure of IOP control after Phaco-GSL surgery [20, 26]. If the postoperative IOP is still high, antiglaucoma medications can be added to control the IOP. If these drugs still cannot control the IOP, glaucoma filtration surgery, such as trabeculectomy, should be considered for these patients [27].

Both study groups showed a significant reduction in IOP and the number of medications from baseline to six and twelve months; however, Phaco-GSL had an apparent advantage over Phaco-alone in separating PAS. Both procedures had equally low postoperative complication rates [16]. Phaco-GSL surgery for treatment of angle-closure glaucoma has no adverse effect on the patients, so it is advisable to routinely perform combined surgery for all patients with angle-closure glaucoma. However, it has also been found in clinical practice that the chamber angle opens well after GSL, but the IOP is still not well-controlled, which may be due to the poor function of the trabecular meshwork that is behind the PAS. Sihota et al. found that chronic PACG eyes had an altered trabecular architecture with fewer spaces and fused trabecular beams, even in areas without PAS [26]. The outflow via the trabecular meshwork may not be compromised in all cases [21]. Kameda et al. reported that the probability of treatment success for all 109 eyes was 85.9% with a mean follow-up of 40 months after Phaco-GSL [28]. Teekhasaenee et al. determined an absolute success rate of 90.4% after Phaco-GSL in eyes with acute angle-closure during a mean follow-up of 20.8 months [10]. With longer observation time, the PAS may reappear leading to the recurrence of IOP increases, although it has been reported that IOP lowering can be maintained over three years after Phaco-GSL surgery [28]. Therefore, patients need to be followed up regularly to check the IOP, anterior chamber depth, and chamber angle.

VGP is an alternative method that does not use a surgical instrument to mechanically remove the PAS but instead injects a cohesive viscoelastic into the angle following IOL implantation. In the present meta-analysis, two papers adopted this VGP approach [12, 19], whereas the remaining six papers utilized mechanical goniosynechialysis (MGSL) [13, 15, 16, 20–22]. Injection of high-molecular-weight viscoelastics near the angle and positive flushing pressure can also significantly resolve PAS, especially in areas where the adhesion is weak [29]. The reason for high IOP values after removal of all PAS is related to the trabecular meshwork. If the trabecular meshwork function is impaired, even if the PAS is resolved, the aqueous drainage outflow is still reduced [19]. Moreover, a previous study has found that subnormal trabecular meshwork function is caused by a loss of trabecular cells and an irregular trabecular architecture, not only where PAS is present, but also in areas not visibly affected by PAS [23]. Therefore, it is possible to encounter patients with poor IOP control after surgery. Although previous studies have reported that VGP can also significantly remove PAS, lower the IOP, and reduce the required antiglaucoma medication dose and that VGP seems to be a much safer procedure than synechialysis which uses a knife or blunt spatula [12, 19], the present study did not compare VGP with MGSL due to the lack of sufficient data for a meta-analysis.

Compared with Phaco-alone, Phaco-GSL may cause more anterior chamber inflammation and complications such as anterior chamber fibrinous exudation, mild-to-severe haemorrhage from the iris or trabecular meshwork, iridodialysis, iris relaxation, irregularly shaped pupils, and transient IOP elevation in the immediate postoperative period. However, the incidence of these conditions is very low, and the anterior chamber inflammatory response subsides quickly with anti-inflammatory eye drops. The time to perform a GSL operation is not very long, and the procedure is not a significant burden on the patient. Razeghinejad et al. found no serious complications in a series of patients who underwent Phaco-GSL [30]. Husain et al. reported that both procedures had equally low postoperative complication rates [16]. Angmo et al. determined that both Phaco-alone and Phaco-GSL groups had comparable results with minimal or no complications [15]. Therefore, Phaco-GSL is a relatively quick procedure and is safely performed by most cataract surgeons.

There are some limitations to this study. First, all included studies had small sample sizes. Second, we did not examine the potential publication bias of the two examined interventions via funnel plots because no more than 10 studies were included in our meta-analysis. Third, there are only a few studies on changes in IOP, number of medications, and PAS extent from preoperative to 12-month postoperative conditions. The results of this meta-analysis should be confirmed in the future based on a higher number of studies. Fourth, the aqueous outflow facility may better reflect differences between the two groups regarding the functional recovery of the chamber angle. Unfortunately, we were not able to perform a subgroup analysis for these variables due to the lack of data. Finally, intra- and postoperative complications, recurrence rate of PAS, and maintenance time of a normal IOP after surgery are also parameters of great significance for the evaluation of both surgical approaches. However, we were unable to find sufficient data to investigate these parameters. Therefore, longitudinal in-depth RCTs with large sample sizes evaluating the aforementioned parameters are required in the future to investigate differences between the two study groups.

## 5. Conclusion

In conclusion, this meta-analysis provides sufficient evidence that Phaco-GSL is advantageous for the extent of PAS compared with Phaco-alone at the 12-month follow-up but not the reduction in IOP and number of antiglaucoma medications. Goniosynechialysis can be safely and quickly performed by most cataract surgeons during phacoemulsification surgery, and it has a long-term effect on the reduction in PAS. Therefore, Phaco-GSL can be considered for the treatment of patients with angle-closure glaucoma and cataract.

## Figures and Tables

**Table 1:** Summary of the characteristics of the included studies.

Author (year)	Study location	Study design	Type of glaucoma	No. of eyes		Mean age (years)		Sex (male/female)		Technique	Follow-up period
				Phaco-GSL	Phaco-alone	Phaco-GSL	Phaco-alone	Phaco-GSL	Phaco-alone		(months)
Varma/2010 [12]	UK	RCT	CACG	25	25	72.4 ± 8.9	72.96 ± 7.8	16/9	8/17	VGP	12
Moghimi/2015 [19]	Iran	RCT	PACG	45	46	61.6 ± 8.3	63.2 ± 6.9	19/26	19/27	VGP	12
Shao/2015 [20]	China	RCT	PACG	23	12	73.61 ± 8.44	69.85 ± 8.56	NR	NR	MGSL	6
Tun/2015 [13]	Singapore	RCT	PACG	11	11	66.75 ± 6.53	67.77 ± 5.18	3/8	2/9	MGSL	12
Xu/2017 [22]	China	RCT	PACG	46	50	53.62 ± 6.43	54.73 ± 6.58	28/18	30/20	MGSL	6
Rodrigues/2017 [21]	UK	RCT	PACG PAC	14	10	67.2 ± 8.4	66.1 ± 7.4	5/9	5/5	MGSL	6
Husain/2019 [16]	Singapore/Vietnam/Thailand/Hong Kong	RCT	PACG PAC	38	40	68.1 ± 9.2	67.3 ± 8.6	13/25	11/29	MGSL	12
Angmo/2019 [15]	India	RCT	PACG	34	30	56.50 ± 9.17	58.77 ± 8.14	NR	NR	MGSL	6

CACG: chronic angle-closure glaucoma; MGSL: mechanical goniosynechialysis; NR: not reported; Phaco-alone: phacoemulsification alone; PAC: primary angle-closure; PACG: primary angle-closure glaucoma; Phaco-GSL: phacoemulsification with goniosynechialysis; RCT: randomized controlled trial; UK: United Kingdom; VGP: viscogonioplasty.

## Data Availability

The data used to support the findings of this study are available from the corresponding author upon request.
